# Thyrotropin/growth hormone co-secreting pituitary adenoma with pancytopenia: a case report

**DOI:** 10.1186/s13256-026-06160-2

**Published:** 2026-06-20

**Authors:** Jie Liu, Yanhui Zhu, Yu Wei, Lei Yu, Yu Li, Qiong Wang, Langen Zhuang, Guoxi Jin, Xiaoyan Pei

**Affiliations:** Department of Endocrinology, The First Affiliated Hospital of Bengbu Medical University, No. 287 Changhuai Road, Bengbu, Anhui 233000 People’s Republic of China

**Keywords:** Pituitary neoplasms, Thyrotropin, Growth hormone, Octreotide

## Abstract

**Background:**

Thyroid-stimulating hormone (TSH)-secreting pituitary adenoma (TSHoma) is a rare functional pituitary tumor that secretes TSH independently, thus stimulating the synthesis and secretion of thyroid hormone. Its clinical manifestation is central hyperthyroidism, and it is easily misdiagnosed as Graves’ disease. At present, the treatment of TSHoma includes surgery, medication, and radiotherapy, and transsphenoidal pituitary surgery remains the first-line treatment.

**Case presentation:**

This case involved a 61-year-old Chinese male with clinical manifestations of weight loss, heart palpitations, and shortness of breath, both thyroid hormone and TSH are elevated. The patient was diagnosed with a TSH/growth hormone (GH) co-secreting pituitary adenoma by serological examination, magnetic resonance imaging of the pituitary gland, and related functional tests. Due to thrombocytopenia and heart disease, the patient was deemed a poor candidate for immediate surgery and was therefore treated with octreotide. Thyroid function tests normalized after medication, and the diagnosis of TSHoma was verified.

**Conclusion:**

Although TSHoma is rare, it is not difficult to diagnose based on clinical presentation, ancillary examination, and functional testing. Thyroid hormone resistance syndrome is very similar to it and is therefore easily misdiagnosed. Early diagnosis and appropriate treatment can effectively control the disease and even achieve a clinical cure.

## Background

Pituitary adenomas are common tumors in the sellar region, with an annual incidence of 3.9–7.4/100,000. The prevalence rate of clinically symptomatic pituitary adenomas is about 1/1000 [1]. Of the pituitary adenomas that cause clinical symptoms, 30% are nonfunctional adenomas (non-hormone secreting), 53% are prolactinomas, 12% are GH-secreting tumors, and 4% secrete adrenocorticotropic hormone in excess. Thyrotropin-secreting tumors account for only 1%, of which 75% are macroadenomas (diameter ≥ 10 mm) [2]. TSHoma is one cause of central hyperthyroidism. Serological examination shows that the levels of free triiodothyronine (FT3) and free thyroxine (FT4) are elevated, but the level of TSH is not appropriately suppressed. Pituitary magnetic resonance imaging can identify these tumors. In recent years, with continuous improvement in laboratory detection technology and the widespread application of magnetic resonance imaging technology, the detection and diagnosis rate of TSHoma has increased significantly, but because the clinical manifestations are similar to those of Graves’ disease, TSHoma can be misdiagnosed resulting in initiation of inappropriate treatment, thus aggravating the disease. It is therefore necessary to discuss the diagnosis and treatment of TSHoma.

## Case presentation

The patient, a 61-year-old Chinese male, presented with unexplained weight loss of approximately 20 kg over the past 4 years, and palpitations and shortness of breath over the last 6 months, accompanied by hand tremor. Thyroid function testing revealed elevated levels of the following: FT3 27.34 pmol/L (2.77–6.50 pmol/L), FT4 99.32 pmol/L (10.43–24.32 pmol/L), TSH 6.37 mIU/L (0.40–4.34 mIU/L), and sex hormone binding globulin (SHBG) > 200 nmol/L (14.50–48.40 nmol/L). Thyroglobulin antibody, thyroid peroxidase antibody, and thyrotropin receptor antibody titers were negative. The patient had no family history of thyroid disease. Physical examination showed emaciation, pallor, arrhythmia, goiter, no exophthalmos or pretibial mucinous edema, and normal muscle strength of the extremities.

**Fig. 1:**
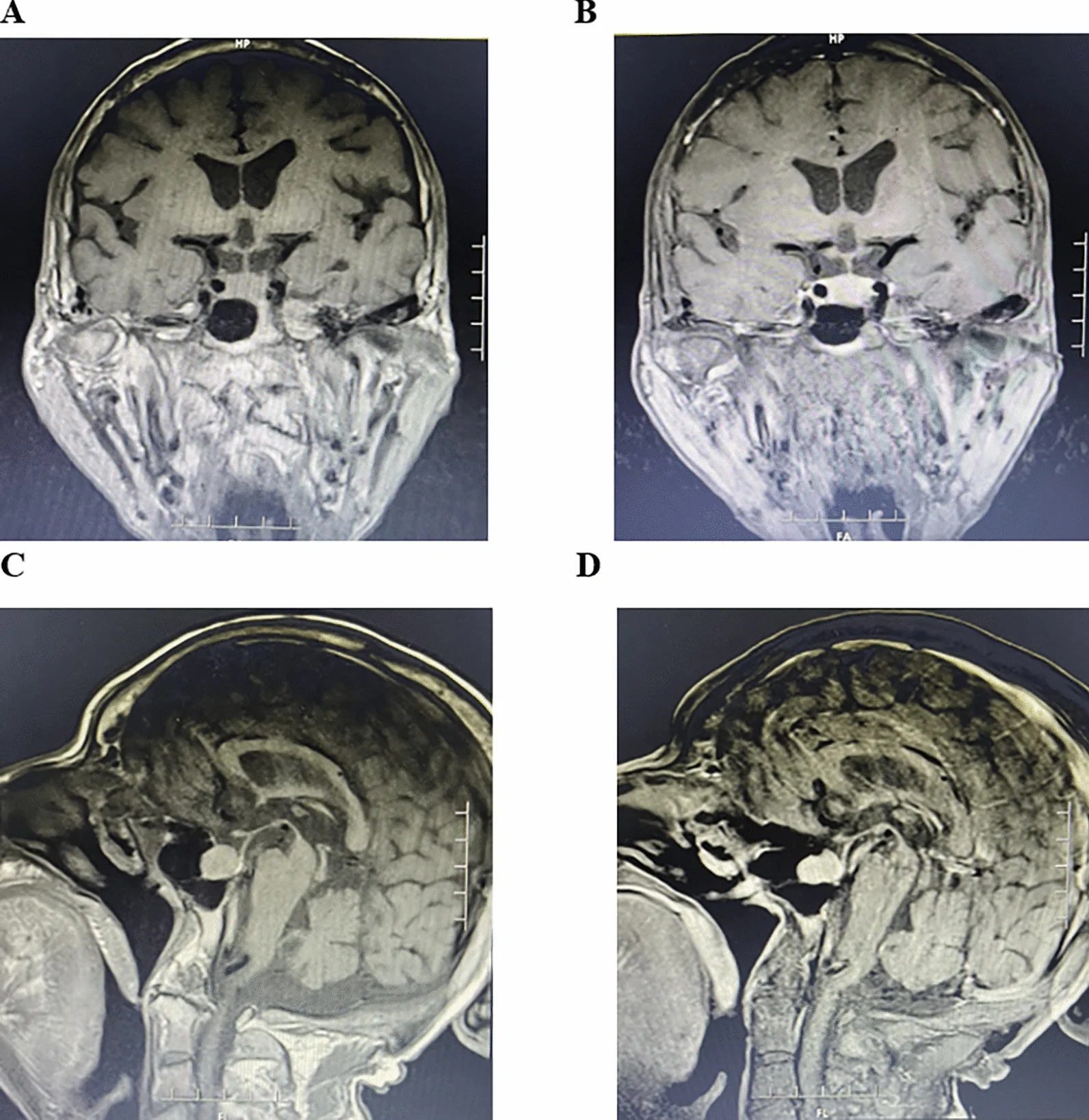
**A** Precontrast T1-weighted coronal slices magnetic resonance imaging (MRI) image. **B** Postcontrast T1-weighted coronal slices MRI image. **C** Precontrast T1-weighted sagittal slices MRI image. **D** Postcontrast T1-weighted sagittal slices MRI image

In addition, FT3 and FT4 levels were elevated, TSH levels were not inhibited, and thyroid autoantibodies were negative; primary hyperthyroidism was therefore excluded. Combined with the results of his physical examination, TSHoma was highly suspected. Pituitary magnetic resonance imaging (Fig. 1) revealed a round mass approximately 15 mm × 16 mm × 14 mm in size, and a dynamic contrast-enhanced scan showed mild enhancement. Thyroid color doppler ultrasonography revealed diffuse bilateral thyroid enlargement with increased blood flow and bilateral thyroid nodules (C-TIRADS 2–3). To establish a definitive diagnosis, an octreotide inhibition test was performed (Table 1), and after injection of octreotide, the TSH inhibition rate of the patient was as high as 91.38%. The diagnosis of a TSH-secreting pituitary tumor was thereby confirmed. The patient’s GH level was elevated at 5.78 ng/ml (< 5.00 ng/ml). Although the patient had no clinical evidence of acromegaly or gigantism, the possibility of GH co-secretion could not be discounted. We administered an oral glucose tolerance test (Table 2), and the patient's GH trough value remained greater than 1 µg/L; thus, GH failed to suppress, and our patient was presumed to possess a TSH/GH co-secreting pituitary adenoma. An electrocardiogram showed atrial fibrillation with rapid ventricular rate, consistent with thyrotoxic heart disease generated by high levels of thyroid hormone.

**Table 1:** Results of octreotide-inhibition test

Variable	0 h	2 h	4 h	6 h	8 h	24 h	IR (%)
FT3 (pmol/L)	26.03	24.97	23.82	21.55	19.87	11.85	–
FT4 (pmol/L)	72.91	74.97	68.86	56.29	50.38	48.84	–
TSH (mIU/L)	4.87	3.32	2.59	1.98	1.73	0.42	91.38

Abbreviations: FT3, free triiodothyronine; FT4, free thyroxine; IR, inhibition ratio: (basal TSH − minimal TSH)/basal TSH × 100%; TSH, thyroid stimulating hormone

**Table 2:** Results of oral glucose tolerance test

Variable	0 h	0.5 h	1 h	2 h	3 h
Glucose (mmol/L)	3.78	6.25	7.32	6.28	–
Insulin (mIU/L)	< 2.00	16.10	31.20	28.10	19.50
GH (ng/ml)	5.70	5.84	4.75	2.77	3.37

Abbreviations: GH, growth hormone

Serological examination of the patient showed that the numbers of red blood cells, white blood cells, platelets, and albumin were significantly reduced and the level of thyroid hormone was high, increasing the risk of surgery. Therefore, the patient was provided with supportive treatment that included leukocyte and platelet transfusions and nutritional support. Metoprolol, 25 mg twice daily per oral, controlled the ventricular rate, propylthiouracil, 50 mg three times per day per oral, alleviated hypermetabolic symptoms. A single intramuscular injection of 20 mg of octreotide diminished TSH and thyroid hormone levels. Three weeks later, the levels of FT3, FT4, and TSH had decreased significantly (Table 3); and routine blood and biochemical tests trended toward normal. Three months later, the levels of FT3, FT4, and TSH were noted to increase (Table 3), while the levels of red blood cells, white blood cells, and platelets decreased. Platelet numbers were as low as 60 × 109/L. Echocardiography showed atrial enlargement, massive tricuspid regurgitation, moderate pulmonary hypertension, and a 50% ejection fraction. The pituitary mass, however, did not shrink. Due to the patient's unstable medical condition, the risk of surgical bleeding, and the high risk of a thyrotoxic crisis during and after surgery, surgical treatment was again postponed. Conservative medical treatment with octreotide and active symptomatic treatment of leukopenia and thrombocytopenia were continued. Surgery would only be considered after the patient’s condition had stabilized.

**Table 3:** Results of thyroid function testing after treatment

Variable	Before treatment	Three weeks later	Three months later	Normal range
FT3 (pmol/L)	27.34	6.44	7.77	2.77–6.50
FT4 (pmol/L)	99.32	19.68	36.30	10.43–24.32
TSH (mIU/L)	6.37	3.00	7.62	0.40–4.34

Treatment: a single intramuscular injection of 20 mg of octreotide and oral administration of 50 mg of propylthiouracil three times a day

FT3, free triiodothyronine; FT4, free thyroxine; TSH, thyroid stimulating hormone

## Discussion

TSHoma primarily occurs in middle-aged or older patients, the incidence in males and females is similar, most patients are asymptomatic at the onset of the disease, and progression is slow [3]. TSHoma is also of monoclonal origin. No mutations of oncogenes or tumor suppressor genes have been uncovered, and the pathogenesis of the disease is unclear [4]. The clinical manifestations of TSHoma are principally divided into the following three aspects: (1) Thyrotoxicosis is caused by excessive secretion of TSH. TSHoma secretes TSH independently and is not regulated by the hypothalamic-pituitary-thyroidal axis, resulting in an increase in thyroid hormone secretion; this causes typical symptoms of thyrotoxicosis that include palpitation, hyperhidrosis, weight loss, goiter, and nodules. Exophthalmos and pretibial myxedema are rare. Significant fluctuations in TSH levels can also infrequently induce Graves’ disease and lead to corresponding symptoms [5]. Our patient exhibited palpitations, shortness of breath, weight loss, and diffuse thyroid enlargement with nodules that may have resulted from enhanced TSH stimulation. In addition, the patient had symptoms of thyrotoxic heart disease such as atrial enlargement and atrial fibrillation; (2) Other anterior pituitary hormones are often oversecreted. Approximately 30% of TSHomas are mixed adenomas [6], and they can also secrete GH, prolactin, corticotropin, and gonadotropin. The most common co-secreted pituitary hormones are GH and prolactin, and co-secretion can lead to acromegaly or gigantism and amenorrhea-galactorrhea syndrome. This may be due to the fact that the cells secreting these hormones jointly express pituitary specific transcription factor-1 (PIT-1) [7]. In our patient, the elevated GH level and the failure of GH inhibition confirmed the diagnosis of a TSH/GH co-secreting pituitary adenoma; and (3) There may be a tumor mass effect. A macroadenoma can compress and infiltrate the pituitary and surrounding tissues and can cause headache, visual field defects, and anterior pituitary dysfunction [8, 9]. Our patient did not have symptoms caused by tumor compression or infiltration.

The diagnosis of TSHoma depends on the comprehensive analysis of clinical manifestations, laboratory and imaging examinations, and functional testing. Thyroid function tests should be conducted repeatedly so as to exclude the presence of interfering antibodies in the serum, different stages of thyroid disease, or the effects of drugs [10]. After excluding all types of interference, if FT3 and FT4 remain elevated and TSH is not suppressed, the possibility of TSHoma should be considered. Although thyroid hormone resistance syndrome (RTH) is principally caused by mutations in the gene encoding the thyroid hormone receptor [11] and exhibits the same clinical and serological characteristics as TSHoma, most patients with RTH have a family history. An increase in SHBG, C-terminal telopeptides of type I collagen, serum glycoprotein hormone alpha-subunit (α-GSU), or α-GSU/TSH further indicate TSHoma [4, 8, 9]. In addition, there are cases of TSHoma complicated by RTH [12]. With genetic testing, we detected no abnormality in our patient. Attention should be paid to the measurement of other anterior pituitary hormones to determine whether there is hormone co-secretion or evidence of pituitary hypofunction. Pituitary magnetic resonance imaging confirms the presence and size of a tumor and its relationship with adjacent structures. Functional testing can additionally be implemented for differential diagnosis. In TSHoma patients, TSH is not stimulated in a thyrotropin-releasing hormone stimulation test nor is TSH inhibited in an L-triiodothyronine inhibition test, while TSH is inhibited in the octreotide inhibition test, contrary to the situation in RTH patients [6].

The primary purpose of the treatment of TSHoma is tumor removal or control of tumor growth, alleviation of any space-occupying effect, restoration of pituitary function, and return of normal functioning of the hypothalamic-pituitary-thyroidal axis. Surgery is the first-line treatment, and adenoma resection via the sphenoidal sinus or sub-frontal route is the first choice [9]. The size, texture, and invasion of the tumor affect the resection rate, which directly affects the postoperative remission rate; however, the endoscopic extended transsphenoidal approach can improve the resection rate [3]. In addition, preoperative preparation is extremely important. When the symptoms of thyrotoxicosis are obvious, the first-line somatostatin analogue (SSA) and the second-line antithyroid drugs should be administered to normalize the levels of free thyroid hormone and to prevent thyroid crisis during and after surgery [13]. SSA normalizes TSH levels in approximately 90% of patients, thyroid hormone levels in 70% of patients, and tumor size in 50% of patients by at least 20% [14, 15]. Antithyroid drugs can only be used for a short time, but they can be used in combination with β-blockers to improve hypermetabolism [16]. Use of antithyroid drugs for an extended period of time will lead to tumor enlargement and hardening and deterioration of pituitary and thyroid function. Such drugs can be taken by patients who are unable to undergo surgery or whose postoperative result is not favorable. High expression of somatostatin receptor (SSTR) on TSHoma cells, especially SSTR2 and SSTR5, is the preferred target for long-acting SSAs [17, 18]. SSA is safe and can also be taken by pregnant women [19], although adverse reactions include gastrointestinal discomfort, cholecystitis, cholecystolithiasis, and hyperglycemia [20, 21]. The dopamine D2 receptor is also expressed on TSHoma cells, but dopamine receptor agonists are more suitable for patients with TSH/GH or TSH/prolactin co-secreting pituitary adenomas [22]. For patients with surgical and drug contraindications or postoperative tumor residual, radiotherapy can be selected; these include general radiotherapy and radiosurgery (such as gamma knife or cyber knife). However, radiotherapy may lead to pituitary dysfunction and necessitate hormonal replacement therapy [23]. Because our patient’s platelet count was very low and he suffered from heart disease, he was not considered a candidate for surgical treatment. FT3, FT4, and TSH decreased significantly after octreotide treatment, which again confirmed the diagnosis of TSHoma. It is worth noting that thyrotoxicosis combined with peripheral blood trilineage cell abnormalities is not uncommon in clinical practice, and studies have found that leukopenia is more common in patients with untreated thyrotoxicosis, with the incidence of granulocytopenia ranging from 36% to 48.81% [24], the incidence of anemia is 22% [25], the incidence of thrombocytopenia is 11.1% [26], and the incidence of pancytopenia is less common [27]. Peripheral blood cell abnormalities can occur before and after treatment of thyrotoxicosis, and some patients may seek medical attention with blood cell abnormalities as the initial symptom and be diagnosed with Graves’disease. After the control of thyrotoxicosis, the blood cell abnormalities in these patients also improve. The mechanism of thyrotoxicosis leading to blood cell abnormalities is unclear and is generally thought to be related to thyrotoxicosis itself and autoimmune reactions: (1) Too much thyroid hormone causes blood vessels in the body's endings to dilate, circulating blood volume increases, and blood is thinned, resulting in a relative decrease in blood cells; (2) A significant increase in thyroid hormone directly inhibits the differentiation of bone marrow hematopoietic stem cells, leading to impaired maturation of blood cells or hindering their release to the periphery [28]; (3) Thyrotoxicosis is a hypermetabolic disease, and the patient's basal metabolic rate increases significantly, resulting in excessive consumption and extreme lack of hematopoietic raw materials; and (4) Patients with thyrotoxicosis produce some autoantibodies against bone marrow hematopoietic cells [29].

## Conclusion

Although TSHoma is a rare disease, in combination with medical history, ancillary examination, and functional testing, its diagnosis is not difficult. We suggest that the physician not instinctively administer antithyroid drugs, thyroid surgery, or radiotherapy. In addition, attention should be paid to whether the patient has hematologic damage, we posit that once the patient is diagnosed with TSHoma, there should follow timely selection of appropriate treatment and close follow-up, a regular review of thyroid function, pituitary magnetic resonance imaging, anterior pituitary hormone levels and hematometry so as to evaluate the status of the disease and thus improve the prognosis.

## Data Availability

Data sharing is not applicable to this article as no datasets were generated or analysed during the current study.

## Abbreviations

TSH: Thyroid-stimulating hormone
TSHoma: Thyroid-stimulating hormone secreting pituitary adenoma
GH: Growth hormone
FT3: Free triiodothyronine
FT4: Free thyroxine
SHBG: Sex hormone binding globulin
PIT-1: Pituitary specific transcription factor-1
RTH: Thyroid hormone resistance syndrome
α-GSU: Serum glycoprotein hormone alpha-subunit
SSA: Somatostatin analogue
SSTR: Somatostatin receptor
